# When Too Much Help is of No Help: Mothers’ and Fathers’ Perceived Overprotective Behavior and (Mal)Adaptive Functioning in Adolescents

**DOI:** 10.1007/s10964-022-01723-0

**Published:** 2023-01-12

**Authors:** İldeniz B. Arslan, Nicole Lucassen, Loes Keijsers, Gonneke W. J. M. Stevens

**Affiliations:** 1 Department of Psychology, Education & Child Studies, Erasmus University Rotterdam, Burgemeester Oudlaan 50, 3000 DR Rotterdam, The Netherlands; 2 Department of Interdisciplinary Social Science, Utrecht University, Padualaan 14, 3584 CH Utrecht, The Netherlands

**Keywords:** Parental Perceived Overprotection, (Mal)Adaptive Functioning, Adolescence, Parental Gender, Longitudinal

## Abstract

Although parental overprotection is theorized to have lasting negative effects throughout a child’s life, there is limited empirical evidence available on its long-term significance on adolescent well-being. This preregistered, three-wave longitudinal study investigated the association of maternal and paternal perceived overprotection in early adolescence with the development of (mal)adaptive psychological, academic, and social functioning throughout adolescence. Data (*N* = 2229; 50.7% girls) from the longitudinal TRacking Adolescents’ Individual Lives Survey (TRAILS) in the Netherlands were used (*M*_age_ T1 = 11.11, T2 = 13.57, T3 = 16.28). At T1, adolescents reported on their mothers’ and fathers’ overprotection. From T1 to T3 adolescents and teachers reported about internalizing problems, academic achievement, prosocial, and antisocial behavior. The results showed concurrent associations between higher levels of perceived overprotection and higher levels of internalizing problems, antisocial behaviors, and (after controlling for parental warmth and rejection) lower levels of academic achievement. Perceived overprotection was positively associated with decreased internalizing problems over time. This longitudinal association disappeared after controlling for baseline levels of internalizing problems, suggesting that this result was less robust than expected. Mothers and fathers did not differ in their associations between perceived overprotection and (mal)adaptive functioning. The findings showed that perceived overprotection is mainly concurrently associated with (mal)adaptive adolescent functioning. Future research recommendations are discussed in terms of stability and bidirectional relations.

## Introduction

It is well-established that parental involvement, support, and protection are related to better wellbeing in adolescents (Khaleque, 2013). Some parents apply such high levels of protection (i.e., overprotection), that it no longer fits with the developmental needs of the child (Bernstein & Triger 2010). Overprotection is theorized to have lasting harmful effects throughout a child’s life, as it may insufficiently prepare adolescents for adulthood through an undermined development of autonomy and feelings of competence (Ungar, 2009). Currently, there is limited research available on the long-term effects of overprotection on adolescent well-being (De Roo et al., 2022). Moreover, some studies report positive associations between too supportive, protective parenting practices and adolescent functioning (e.g., Fingerman et al., 2012), raising the question whether overprotection may relate to better functioning in adolescents, especially for specific domains of functioning. Longitudinal studies, differentiating between fathers and mothers, and including *both* adaptive and maladaptive outcomes of adolescent functioning may explain the heterogeneity of results between studies, but are currently lacking. This preregistered longitudinal study investigates associations between maternal and paternal perceived overprotection in early adolescence (mean age 11) and the development of (mal)adaptive psychological, academic, and social functioning throughout adolescence (11 to 16 years).

### Parental Overprotection and (Mal)Adaptive Adolescent Functioning

Adolescence, referring to the transition from childhood to adulthood, has often been depicted as a period of “storm and stress” (Arnett, 1999); a stage in which many psychological, social, biological, and cognitive developmental changes happen as a result of, for example, increasing mood instability (Maciejewski et al., 2017), or increasing autonomy (Galambos & Costigan, 2003). These changes may have significant implications for different aspects of adolescent development. For example, throughout adolescence, levels of psychological problems, such as internalizing problems, generally tend to increase (Lunetti et al., 2022). Research on social behaviors is less consistent. Antisocial behavior in general shows a peak in mid-adolescence as a result of increasing participation in risk-taking activities (Moffitt, 2006), however, specific antisocial behaviors, such as physical aggression, have been found to decrease throughout adolescence (Bongers et al., 2003). Research on the development of prosocial behavior reported decreases, increases or no changes throughout adolescence (Carlo et al., 2015). On the academic level, different developmental factors, for example school stage (transition from educational levels) (Martin et al., 2015), have been suggested to be related to decreases in academic achievement. Specifically, adolescents may experience the changes in social and learning environment, teachers, and friends as stressful, which may negatively affect their academic motivation and performances (Martin et al., 2015).

Together with certain transitions that take place (i.e., moving to secondary education), adolescence is characterized by a growing desire for autonomy and independence. Over time, adolescents become more independent of their parents during adolescence as they spend an increasing amount of time outside the family context (separation-individuation theory; Blum, 2004). An autonomy supportive environment allows adolescents to grow, to curiously explore their environment and pursue activities that provide challenge and satisfaction (Soenens et al., 2010). In this way, basic psychological needs of adolescents (such as autonomy, competence, and relatedness) are fulfilled, which promotes adolescents’ well-being on various domains of functioning, including psychological, academic, and social functioning (Self-Determination Theory; Ryan & Deci, 2002). The effects may be lasting, as better functioning on these domains allows adolescents to develop skills that are required for success in adulthood (National Academies of Sciences, Engineering, & Medicine, 2020).

Overprotection may negatively affect various domains of adolescent functioning through hampering adolescents’ need for autonomy and feelings of competence. In this study, overprotection is defined as overprotective, anxious parenting with a tendency for control attempts (Young et al., 2013). Overprotective parents try to ensure their child’s safety and well-being by being excessively involved in their child’s daily activities and experiences (De Roo et al., 2022). Regarding psychological functioning, as parental autonomy granting and support for independence facilitates self-efficacy and confidence (McLeod et al., 2007), overprotection may diminish adolescents’ self-efficacy and coping behaviors, thereby increasing psychological problems, such as internalizing problems (Segrin et al., 2013). In terms of academic functioning, autonomy limiting parenting behaviors may hamper adolescent’s own successful time management and decision-making (Lareau, 2011), and reduce intrinsic motivation and increase extrinsic motivation (Schiffrin et al., 2014). Moreover, social functioning may be negatively affected by overprotection. Overprotective parents tend to be more anxious that something will happen to their child and consequently limit their child’s autonomy by forbidding them to do things that their peers are allowed to do (Markus, 2003). In this way, overprotection may elicit more difficulties within peer-relationships, because these adolescents get less opportunities to independently develop healthy relationships. In turn, adolescents may show less positive social behaviors towards their peers, such as prosocial behaviors (i.e., helping, sharing and comforting; Eisenberg et al., 2015). Overprotection (through prohibition and disapproval of friendships) could also be linked to negative social behaviors, such as antisocial behavior (e.g., Keijsers et al., 2012). Together, a large amount of empirical studies support the abovementioned notion that overprotection may hinder psychological, academic, and social functioning in adolescents.

Drawing on the background of positive psychology, the Two-Continua Model of Keyes (Keyes, 2014) stresses that the absence of problems is not equivalent to better functioning. Rather, functioning refers to a multidimensional concept of “a state of complete physical, mental and social functioning” (World Health Organization, 2006) based on two dimensions: The absence or presence of negative outcomes and the absence or presence of positive outcomes. A better picture of overall functioning is retrieved by examining the wide range of maladaptive and adaptive dimensions of functioning (Arslan et al., 2021; Westerhof & Keyes, 2010). Hence, this study will address adolescent functioning in terms of maladaptive (i.e., internalizing problems, antisocial behavior) and adaptive (i.e., academic achievement, prosocial behavior) functioning.

One open question in the literature is whether overprotection might in fact also be related to better functioning, especially for adolescents’ academic functioning. A small proportion of studies found that sometimes too much parental support, involvement or protection were related to positive functioning, such as better academic achievement and more motivation at school (e.g., Fingerman et al., 2012). Different explanations have been suggested for these positive associations. For instance, helicopter parenting (i.e., overinvolved, overprotective parenting) can occur within warm or highly controlled family contexts, and parts of associations between helicopter parenting and child adjustment may be explained by the level of other positive or negative parenting behaviors that children experience (Rote et al., 2020). In this study it will be taken into account that overprotection may be differentially related with adolescent functioning when the level of parental warmth and rejection are accounted for.

### Mothers’ and Fathers’ Overprotection

There is an ongoing debate on whether mothers and fathers are different or similar in their parenting roles (Majdandžić et al., 2018). Some scholars state that mothers and fathers are generally assumed to differ in their input and contributions to their child’s development (Paquette, 2004). Fathers may have more of a function of opening children to the outside world through physical and challenging play, while mothers are more care-oriented and soothing. Other scholars argue that mothers’ and fathers’ parenting roles do not differ from each other (Fagan et al., 2014). A recent systematic review on overprotection suggested that there may be differences in associations between maternal and paternal overprotection and adolescent maladaptive functioning, as the effects of maternal overprotection were larger compared to paternal overprotection (De Roo et al., 2022). These studies predominantly focused on maladaptive functioning. As such, it remains an open question whether effects of maternal and paternal overprotection differ with regard to adaptive functioning.

## Current Study

Through a multidimensional approach with a focus on both maladaptive and adaptive adolescent functioning, addressing overprotection of both fathers and mothers, this study aims to better understand some of the heterogenous findings on how overprotection relates to adolescent functioning. This preregistered three-wave study examined the longitudinal relation between maternal and paternal overprotection (as perceived by adolescents) and the development of psychological functioning (represented by internalizing problems), academic functioning (represented by academic achievement), and social functioning (represented by prosocial behavior and antisocial behavior) in adolescents. The first research question entailed how levels of mothers’ and fathers’ perceived overprotection at age 11 are related to (a) the initial level of and (b) the change in adolescent outcomes from age 11 to 16. Perceived overprotection is expected to be related to higher initial levels of internalizing problems (hypothesis 1a), lower initial levels of prosocial behavior (hypothesis 2a), and higher initial antisocial behaviors (hypothesis 3a) as well as stronger increases in internalizing problems (hypothesis 1b), stronger decreases in prosocial behavior (hypothesis 2b), and stronger increases in antisocial behavior (hypothesis 3b) between age 11 to 16. No a priori hypotheses were formulated regarding academic achievement. The second research question pertained to the exploration of parental gender differences in the associations between perceived overprotection and (a) the initial level of and (b) the change in adolescent outcomes. This was answered without a priori hypotheses. This study was preregistered at the Open Science Framework (https://osf.io/r65ns/?view_only=02eb52d59001476ab61fcf63041fd9eb).

## Methods

### Participants and Procedure

Participants were selected from a Dutch multidisciplinary, multi-wave, and multi-informant prospective cohort study: TRAILS (TRacking Adolescents’ Individual Lives Survey) (Oldehinkel et al., 2015). Participants were initially selected from five municipalities in the north of the Netherlands (both urban and rural areas). Simultaneously, primary schools were approached. Participants were included when their schools were willing to participate in the study. Adolescents filled out the questionnaires at school, under supervision of research assistants. Teachers also filled out several questionnaires about these adolescents. Parents filled out questionnaires and were interviewed by research assistants. Detailed information about the study and data collection procedures can be found in previous manuscripts (Oldehinkel et al., 2015; Ormel et al., 2012).

In the current paper, the first three of six waves of the ongoing study were used: Time 1 (T1; 2001–2002; *M*_age_ = 11.11, *SD*_age_ = 0.56), Time 2 (T2; 2003–2004; *M*_age_ = 13.57, *SD*_age_ = 0.53), and Time 3 (T3; 2005–2007; *M*_age_ = 16.28, *SD*_age_ = 0.71). The total sample in this study consisted of 2229 participants (50.7% girls), with different sample sizes based on different time points and measures (see Table 1). The smallest effect size of interest (SEOI) was 0.10 (according to Cohen’s rules of thumb). To detect such small effects, with 80% power (two-tailed, alpha = 0.05) we needed a minimum of 779 participants. Hence, the dataset was sufficiently powered to find small effects.

**Table 1:** Descriptives and Correlations for the Measures of Interest

	*M*(*SD)*	Range	*N* ^a^	Overprotection	Adolescent Functioning	
				T1	T1	T2
Overprotection (T1)	1.86(0.38)	1.00–3.50	2205			
Internalizing Problems (T1)	0.36(0.24)	0.00–1.42	2201	0.30^**^		
Internalizing Problems (T2)	0.33(0.24)	0.00–1.48	2093	0.17^**^	0.50^**^	
Internalizing Problems (T3)	0.32(0.25)	0.00–1.71	1660	0.14^**^	0.41^**^	0.59^**^
Academic Achievement (T1)	3.62(0.90)	1.00–5.00	1918	−0.08^**^		
Academic Achievement (T2)	3.32(0.78)	1.00–5.00	1469	−0.05	0.33^**^	
Academic Achievement (T3)	3.18(0.75)	1.00–5.00	891	−0.02	0.22^**^	0.38^**^
Prosocial Behavior (T1)	3.35(0.67)	1.18–5.00	1927	−0.08^**^		
Prosocial Behavior (T2)	3.23(0.64)	1.36–5.00	1391	0.01	0.38^**^	
Prosocial Behavior (T3)	3.22(0.67)	1.27–5.00	901	0.03	0.35^**^	0.32^**^
Antisocial Behavior (T1)	0.35(0.37)	0.00–2.88	2206	0.17^**^		
Antisocial Behavior (T2)	0.29(0.33)	0.00–2.72	2084	0.10^**^	0.54^**^	
Antisocial Behavior (T3)	0.23(0.30)	0.00–3.12	1657	0.16^**^	0.37^**^	0.50^**^
Covariates						
Rejection (T1)	1.48(0.31)	1.00–3.47	2205	0.43^**^		
Warmth (T1)	3.21(0.50)	1.17–4.00	2206	0.17^**^		
SES (T1)	−0.05(0.80)	−1.94–1.73	2187	−0.09^**^		
Age Adolescent (T1)	11.11(0.56)	10.01–12.58	2229	−0.01		

^a^ At T1, the primary school teacher reported on academic achievement and prosocial behavior. At T2 and T3, the mentor filled out the questionnaires as the adolescents have different teachers in secondary school. Hence, response rates for these measures differed throughout the measurement waves, with more missing cases at T2 and T3

^**^p < 0.01

In terms of parental educational level, 6.8% of the mothers and 5.7% of the fathers had a low education level (finished elementary education), 66.4% of the mothers and 59.1% of the fathers had a medium educational level (finished lower or higher tracks of secondary education), and 26.8% of the mothers and 35.2% of the fathers were highly educated (finished senior vocational education or university). Of the total sample, 74.2% of the parents were married and lived together, 13.1% were divorced, 8.3% were never married, 1.4% were widowed, and 1.0% was married but did not live with their spouse. In total 89.4% of the sample consisted of adolescents from which both parents were born in the Netherlands, and for 10.6% at least one parent was born in another country than the Netherlands (i.e., Turkey, Morocco, Suriname, Netherlands Antilles, Indonesia or Maluku, or another country).

### Measures

#### Perceived Overprotective Parenting

Adolescents reported overprotectiveness of their mothers and fathers at age 11 (T1) through the overprotection subscale of the short version of the Egna Minnen Beträffande Uppfostran (My Memories of Upbringing) for Children (EMBU-C) (Markus, 2003). The 12 items on the overprotection scale cover fearful, anxious, guilt engendering, and intrusive parenting behaviors (e.g., “Does your mother/father forbid you to do things that your classmates are allowed to do because they are afraid of something happening to you?”). The items were rated on a four-point scale (ranging from 1 = *No, never* to 4 = *Yes, always*), for mothers and fathers separately. Cronbach’s alphas in this sample were 0.70 for mothers and 0.71 for fathers. Sufficient support for the factorial and construct validity of the EMBU-C has been established (Markus, 2003), also using the TRAILS data (Deković et al., 2006). The one factor structure for mothers’ and fathers’ perceived overprotection that was demonstrated in earlier studies (e.g., Aluja et al., 2006) was replicated in this sample (see Supplementary Material 1 for the results of the Confirmatory Factor Analyses).

#### Internalizing Problems

Adolescents reported on their own internalizing problems with the Youth Self-Report (YSR; Achenbach and Rescorla, 2001) completed at all three assessments (T1 to T3). A total score of internalizing problems was constructed by taking the mean scores of all items on the following three scales: anxious/depressed behavior (13 items; e.g., “I feel worthless”), withdrawn/depressed behavior (8 items; e.g., “I would rather be alone than with others”), and somatic complaints (10 items; e.g., “I feel overtired”). Adolescents rated each item as 0 *(not true)*, 1 *(somewhat/sometimes true)*, or 2 *(very/often true)*. Cronbach’s alphas in this sample were 0.87 (T1), 0.88 (T2), and 0.89 (T3). The YSR was found to be adequately valid and reliable (Achenbach & Rescorla, 2001).

#### Academic Achievement

Teachers rated adolescents’ achievement (grades), work pace (e.g., “The student shows good work pace”), and effort (e.g., “The student shows good effort”), by means of five items in T1 and nine items in T2 and T3. This teacher questionnaire was developed for TRAILS. The different number of included items in the different waves accommodate for the different number of subjects in the Dutch school system in T1 compared to T2 and T3. The questionnaire was filled out by the primary school teacher at T1, and the secondary school teacher (“mentor”) at T2 and T3. Generally, a mentor also teaches a specific subject, but is assigned to a specific class to keep track of the student’s progress and to provide guidance through additional class hours. Exploratory factor analyses in earlier work on the same dataset (Sijtsema et al., 2014) demonstrated one-factor structures with factor loadings above 0.66 and indicated the use of the instrument for longitudinal analyses. Mean scores on the items were calculated. Cronbach’s alphas in this sample were 0.85 (T1), 0.90 (T2), and 0.90 (T3) and are comparable to previous TRAILS studies (e.g., Verboom et al., 2014).

#### Prosocial Behavior

At T1, T2, and T3, teachers (or mentors) rated adolescents’ prosocial behavior with the Prosocial Behavior Questionnaire (PSBQ) which consisted of eleven items from an earlier prosocial behavior questionnaire (Tremblay et al., 1992) and four items from another study on solidarity (Lindenberg et al., 1998). Teachers rated each item (e.g., “Shows sympathy for someone who has made a mistake”) on a five-point scale (ranging from 1 = *Never* to 5 = *Always*). Mean scores on the items were calculated. Cronbach’s alphas in this sample were 0.93 (T1), 0.92 (T2), and 0.93 (T3), comparable to previous TRAILS studies (e.g., Veenstra et al., 2008).

#### Antisocial Behavior

Adolescents rated their own antisocial behavior on an adapted version of the Antisocial Behavior Questionnaire (ASBQ; Moffit & Silva, 1988) at T1, T2, and T3. The ASBQ consisted of 26 items measured at T1 to T3. Adolescents rated each item (e.g., “Have you ever hit someone at school?”) on a five-point scale (ranging from 0 = *Never* to 4 = *7 times or more*). Mean scores on the items were calculated. Cronbach’s alphas in this sample were 0.86 (T1), 0.85 (T2), and 0.86 (T3), comparable with previous TRAILS studies (e.g., Sentse et al., 2010).

#### Covariates

Several covariates (background family, background adolescent, and parenting constructs) were included in the analyses (see Supplementary Material 2, Table 1 for bivariate correlations between the covariates and adolescent functioning outcomes). Background information about the family and the adolescent, were added as covariates in the main and sensitivity analyses. Background family information (socio-economic status, marital status) was measured through the parent interviews at T1. Family socio-economic status (SES) was measured as the z-standardized mean score of mothers’ and fathers’ occupational position (based on the International Standard Classification for Occupations; Ganzeboom & Treiman, 1996), educational attainment, and family income. The Cronbachs alpha for this scale in this sample was 0.84. Adolescent background information (sex, age, and immigration background as in having parents with a native Dutch or an immigrant background) was measured through self-reports at T1.

The parenting constructs Warmth and Rejection were included as covariates in the sensitivity analyses. Adolescents rated maternal and paternal warmth (e.g., “Does your mother/father make it obvious that they love you?”) and rejection (e.g., “Is your mother/father sometimes harsh and unkind to you?”) at T1 using the subscales Warmth (18 items) and Rejection (17 items) of the EMBU-C (Markus, 2003). Cronbach’s alphas in this sample indicated good reliabilities: 0.91 (mothers) and 0.91 (fathers) for Warmth, and 0.84 (mothers) and 0.84 (fathers) for Rejection.

### Missing Data

In the preregistration, information is provided regarding how the sample size and all measures in the study were determined. All available data (on T1–T3) of the variables of interest was used. To assess the missing data patterns (see Table 1 for amount of missingness) among the study variables and background variables, we computed Little’s Missing Completely at Random (MCAR) test (*χ2*(1541, *N* = 2229) = 2136.60, *χ2/*df = 1.38). As the chi-square test is quite sensitive to sample size, the normed (or relative) approach was applied by dividing the chi-square by the df (Kline, 2016). Even though there is no strict consensus, when this NC (normed chi-square) is below 3 the data and the model (here MCAR) are matching. Thus, with chi/df < 3 the data were missing completely at random and the full information maximum likelihood (FIML) estimation (Schafer, 1997) was applied to include all available data in the analyses. Moreover, all available demographic variables were included in the models as correlates, which is considered as the most effective method in reducing parameter biases in model fit (Graham, 2003) by optimizing the FIML procedure.

### Analyses

**Fig. 1:**
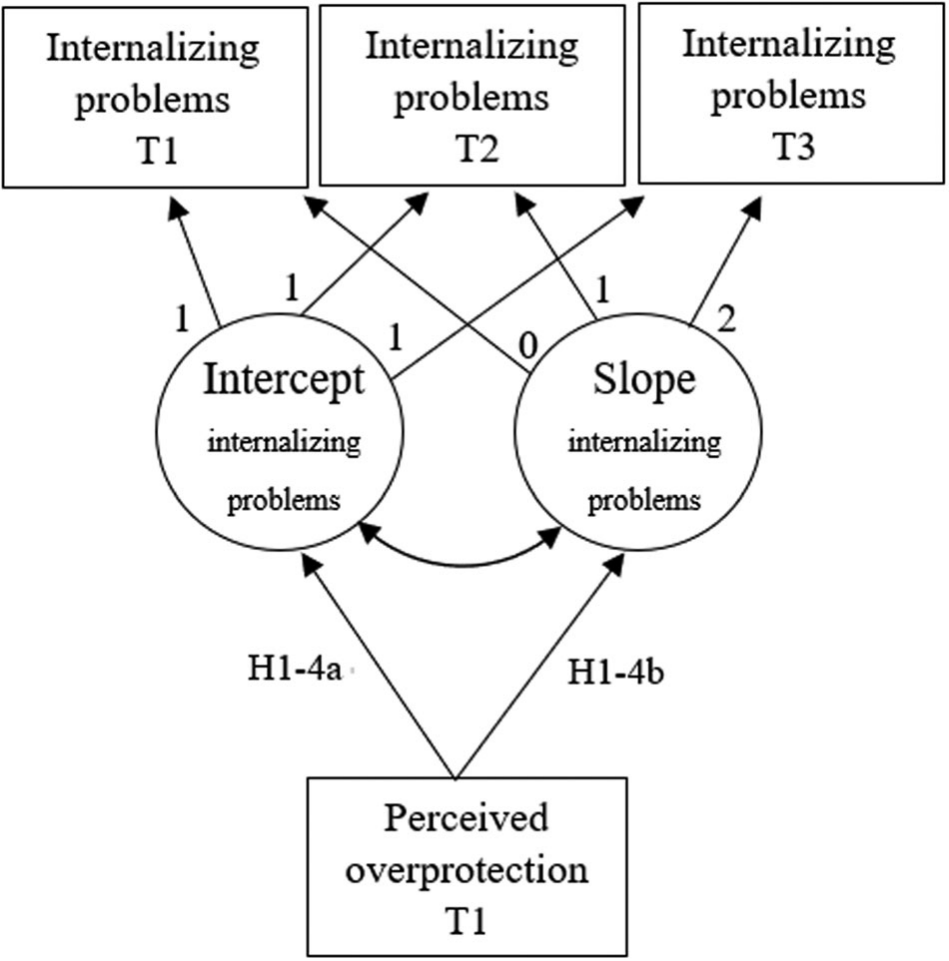
Research question 1: Multivariate latent growth model of perceived parental overprotection and (the intercept and slope factor of) adolescent internalizing problems. T time. Numbers along the arrows represent factor loadings. H hypothesis

To answer the research questions as preregistered, we performed Latent Growth Curve Modeling (Duncan & Duncan, 2004). Data were analyzed in Mplus 8.5 (Muthén & Muthén, 2017). An intercept and linear slope were estimated per outcome measure. These latent growth factors were correlated with perceived overprotection at T1 to test hypotheses 1 to 3 (using a statistical model for internalizing problems illustrated in Fig. 1). The correlation between fathers’ and mothers’ perceived overprotection was >0.70 (*r* = 0.80). To address multicollinearity issues, the analyses for research question 1 were performed with a composite (combined) variable for perceived overprotection of father and mothers.

To answer research question 2, multigroup multivariate latent growth analyses were performed with perceived overprotection by mothers and fathers. With chi-square model fit comparisons, the overall model fit was compared when both paths from perceived overprotection to the intercept and the slope factor of adolescent functioning were freely estimated and constrained to be equal. Differences between the paths of mothers and fathers were confirmed in case of significant difference in fit between the models. Each individual path (to the intercept factor and to the slope factor) was also tested separately to examine parent gender differences more closely. Child and family background characteristics were included as covariates in the models by estimating regression paths with the intercept and slope factors of the outcomes. Sensitivity analyses were performed for research question 1 and research question 2 (as preregistered) by adding the subscales Warmth and Rejection from the EMBU-C.

Because perceived overprotection, internalizing problems, and antisocial behavior were non-normally distributed, Maximum Likelihood with Robust standard errors (MLR) was used. Overall fit of each model was determined by the chi-square (χ^2^), the Comparative Fit Index (CFI), the Tucker-Lewis Index (TLI), and the Root Mean Square Error of Approximation (RMSEA). A good model fit is indicated by CFI and/or TLI values close to 0.950 and not below 0.900, and/or RMSEA values below 0.080 (Brown, 2004).

Hypotheses were confirmed if the regression paths from perceived overprotection to the intercept and/or slope factors of each outcome were significant on a *p* value of < 0.05. In terms of effect sizes, standardized effects were interpreted as suggested by Cohen and expanded by Sawilowsky (2009) (0.01 and lower: very small effect; 0.01–0.20: small effect; 0.20–0.50: medium effect; 0.50–0.80: large effect, 0.80–1.20: very large effect, 1.20–2.00: huge effect). With regard to the slope factors, *SD* change per measurement (T) was used as an indicator of effect size Cohen’s *d*.

## Results

Descriptive information and correlations are presented in Table 1. All univariate linear growth models had sufficient to excellent fit (all models were RMSEA < 0.08, CFI > 0.935, TLI > 0.806; Supplementary Material 2, Table 2). Between ages 11 and 16, mean levels of adolescents’ self-reported internalizing problems and antisocial behaviors, and teacher-reported academic achievement and prosocial behavior decreased (Table 2), but effect sizes were small. The significant variances around the intercept and slope factors (except for prosocial behaviors) indicated differences between individuals in their initial levels and rates of change which allowed us to continue with multivariate models (Duncan & Duncan, 2004) in which the intercept and slope factors (for all but prosocial behaviors) were regressed on parental perceived overprotection (Fig. 1).

**Table 2:** Parameter Estimates of the Intercept and Slope Factors for the Linear Growth Models

	Intercept		Slope			Intercept-Slope Correlation
Model	*M(SE)*	s^2^*(SE)*	*M(SE)*	s^2^*(SE)*	*Effect size*^a^	*r*
Internalizing Problems	0.36(0.01)^***^	0.04(0.003)^***^	−0.03(0.003)^***^	0.01(0.001)^***^	−0.15	−0.33^***^
Academic Achievement	3.59(0.02)^***^	0.29(0.04)^***^	−0.24(0.02)^***^	0.06(0.02)^*^	−0.45	−0.54^***^
Prosocial Behavior	3.34(0.01)^***^	0.18(0.03)^***^	−0.09(0.01)^***^	0.001(0.02)	−0.21	−0.97
Antisocial Behavior	0.35(0.01)^***^	0.09(0.01)^***^	−0.06(0.004)^***^	0.01(0.01)^**^	−0.20	−0.61^***^

^a^ Estimated effect size of change per measurement interval (years); expressed in terms of slope/SD_intercept_, analogues to Cohen’s D change (medium = 0.50, small = 0.20)

^*^p < 0.05; ^**^p < 0.01;^. ***^p < 0.001

### Parental Perceived Overprotection and Adolescent Functioning

**Fig. 2:**
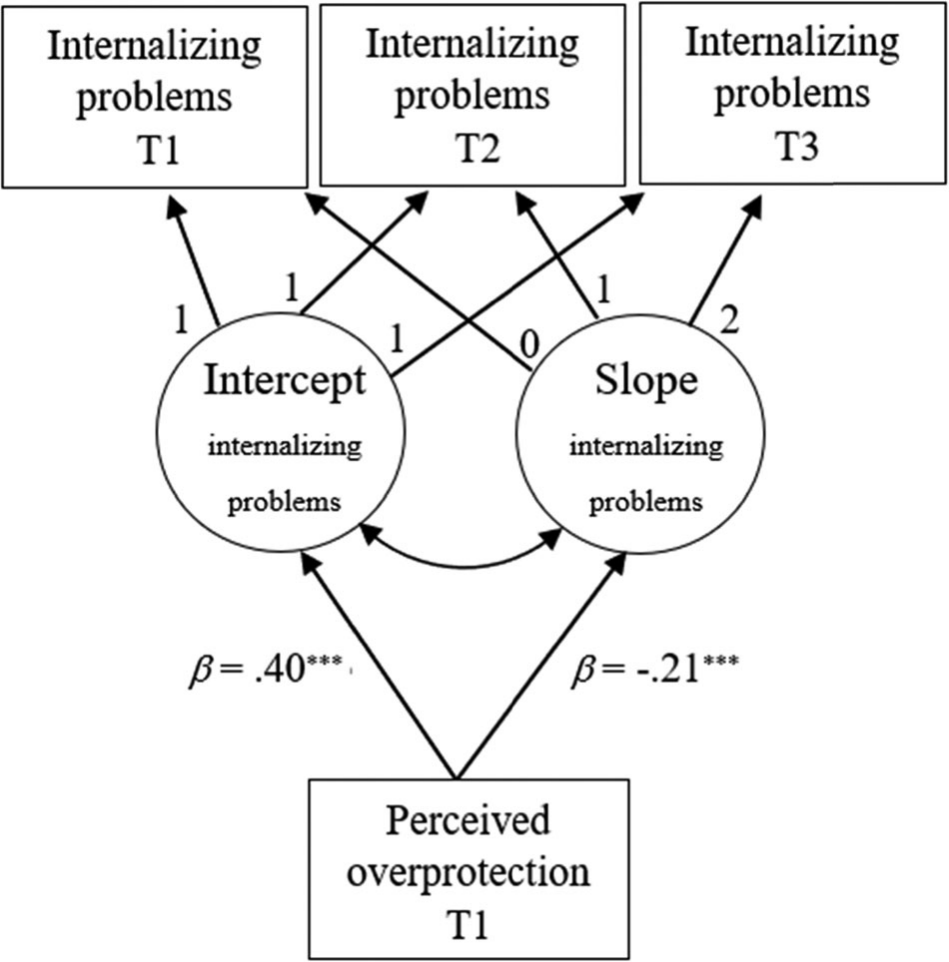
Regression coefficients from the multivariate latent growth model of perceived parental overprotection and adolescent internalizing problems (research question 1). T time. Numbers along the arrows represent factor loadings. All regression coefficients are standardized. ^***^p < 0.001

To answer research question 1, parental perceived overprotection was added as predictor of the estimated intercept (T1 level) and slope (rate of change) of the outcomes^1^. All models showed a good fit (all models were RMSEA < 0.04, CFI > 0.961, TLI > 0.884; Supplementary Material 2, Table 2). As hypothesized (hypothesis 1a), higher parental perceived overprotection was concurrently related to higher initial levels of internalizing problems (Table 3; medium effect; see Fig. 2 for a visualisation of this result). With regard to change, perceived overprotection was associated to an unexpected (hypothesis 1b) stronger decrease in internalizing problems over time (medium effect). For further exploration (not preregistered), in supplementary sensitivity analyses, it was assessed whether this result could have been caused by regression to the mean with regard to internalizing problems. To test this, the slope factor of internalizing problems was regressed on the intercept factor (model showed good fit indices: RMSEA = 0.03[90% CI = 0.02, 0.05], CFI = 0.990, TLI = 0.971). The association between higher levels of perceived overprotection and decreases in internalizing problems over time was no longer significant (*β* = −0.06, *p* = 0.174). In additional analyses adolescent sex differences in initial levels and development of internalizing problems^2^ and their associations with perceived overprotection at T1 were explored. Significant group differences were found for the intercept and slope of internalizing problems (Supplementary Material 2, Table 5). Girls showed higher initial levels of internalizing problems than boys. Girls and boys significantly differed in their development of internalizing problems and antisocial behavior. The negative mean slope of internalizing problems was only significant in boys, indicating that the decrease in internalizing problems was only observed in boys. No significant group differences were retrieved when comparing the overall model fit of the models. However, constraining the relation between perceived overprotection and the intercept factor of the outcomes yielded significant group differences: the relation between higher initial levels of perceived overprotection and higher initial levels of internalizing problems was stronger in girls than in boys. No adolescent sex differences were found when constraining the individual pathway from perceived overprotection to the slope factor of internalizing problems.

**Table 3:** Parameter Estimates of the Regression Paths from Overprotection to (Mal) Adaptive Functioning in Adolescence

	Internalizing Problems		Academic Achievement		Prosocial Behavior		Antisocial Behavior	
	Β (*SE*)	*β*	Β (*SE*)	*β*	Β (*SE*)	*β*	Β (*SE*)	*β*
Preregistered Analyses								
Overprotection → Intercept [hypotheses 1a-3a]	0.20(0.01)^***^	0.40	−0.08(0.05)	−0.06	0.00(0.03)	0.00	0.10(0.02)^***^	0.13
Overprotection → Slope [hypotheses 1b-3b]	−0.05(0.01)^***^	−0.21	0.03(0.04)	0.04	N/A		−0.02(0.01)	−0.05
Sensitivity Analyses (with Covariates Warmth and Rejection)								
Overprotection → Intercept	0.13(0.02)^***^	0.26	−0.15(0.06)^*^	−0.10	−0.04(0.04)	−0.04	0.04(0.02)	0.05
Overprotection → Slope	−0.03(0.01)^**^	−0.11	0.04(0.05)	0.06	N/A		0.02(0.02)	0.05

^*^p < 0.05; ^**^p < 0.01; ^***^p < 0.001

Perceived overprotection was not related to initial levels or the rate of change in adolescents’ academic achievement (no a priori hypotheses), or prosocial behaviors (hypothesis 2a and 2b). Finally, perceived overprotection was positively related with initial levels (small effect; hypothesis 3a), and not related to the change (hypothesis 3b) of antisocial behavior.

Preregistered sensitivity analyses in which parental warmth and rejection were controlled for (all models showed a good fit, see Supplementary Material 2, Table 3), demonstrated similar results for the association between perceived overprotection and (initial levels and change in) adolescent internalizing problems (hypothesis 1a), but a somewhat different pattern for the other outcomes (Table 3). After controlling for warmth and rejection, higher perceived overprotection was related to lower initial levels of academic achievement (small effects). Perceived overprotection was no longer related to initial levels and change in antisocial behavior (hypothesis 3a). Supplementary Material 2, Table 4, shows the regression coefficients covariates warmth and rejection and initial levels and development of the outcomes from the main models with perceived overprotection. Rejection and, mostly, warmth were significantly related to the initial levels and development of almost all adolescent outcomes.

### Parental Gender Differences

To answer research question 2, multigroup multivariate latent growth analyses were performed in which mothers and fathers were compared in their pathways between perceived overprotection and the estimated intercept and the slope of the outcomes. All models showed a good fit (Supplementary Material 2, Table 2). No significant group differences were retrieved when comparing the overall model fit of the models (Table 4). When examining each individual pathway separately, the association between perceived overprotection and the intercept factor of adolescent functioning did not differ for mothers and fathers. No parental gender differences were found when constraining the individual pathway of perceived overprotection to the slope factor of adolescent functioning to be equal.

**Table 4:** Parameter Estimates of the Intercept and Slope Factors for the Linear Growth Model and the Regression Paths from Overprotection to (Mal) Adaptive Functioning in Adolescence, for Mothers and Fathers

	Internalizing Problems	Academic Achievement	Prosocial Behavior	Antisocial Behavior
	Δχ^2^(*df)*^*a*^	Δχ^2^(*df)*^*a*^	Δχ^2^(*df)*^*a*^	Δχ^2^(*df)*^*a*^
Overall Model	0.32(2)	1.24(2)	0.59(1)	0.82(2)
Perceived Overprotection → Intercept	0.22(1)	1.04(1)	0.59(1)	0.52(1)
Perceived Overprotection → Slope	3.09(1)	0.19(1)	N/A^b^	0.53(1)
Sensitivity Analyses (with Covariates Warmth and Rejection)				
Overall Model	0.23(2)	3.98(2)	1.17(1)	0.05(2)
Perceived Overprotection → Intercept	0.01(1)	3.05(1)	1.17(1)	0.03(1)
Perceived Overprotection → Slope	0.21(1)	0.28(1)	N/A^b^	0.004(1)

^a^Comparison of freely estimated model versus constrained model (chi-square difference test)

^b^Because the slope variance of prosocial behavior was not significant, the slope variance was constrained to zero

Sensitivity analyses (all models showed a good fit, see Supplementary Material 2, Table 3) showed that mothers and fathers did not differ in their pathways from perceived overprotection to adolescent functioning, when controlled for maternal and paternal warmth and rejection. Comparing overall model fit (paths freely estimated versus constrained) yielded no significant group differences (Table 4). Mothers and fathers did not differ in their pathways between perceived overprotection and both the intercept factor as well as the slope factor of adolescent functioning.

## Discussion

Normative developmental changes during adolescence require that parents relinquish guidance, protection, and control over their child to promote their child’s healthy functioning later in life. However, certain levels of protection may not be developmentally appropriate, and may frustrate adolescents’ psychological needs and may thus be detrimental for adolescent functioning on various developmental domains. Empirical research demonstrated that overprotection was concurrently linked to maladaptive functioning in adolescents, but longitudinal research is still scarce (De Roo et al., 2022). As such, this study contributed to the knowledge on the role of perceived overprotection in relation to the long-term development of adolescent functioning.

This preregistered three-wave study among 2229 adolescents examined the contemporary and five-year longitudinal associations between fathers’ and mothers’ perceived overprotection and both maladaptive and adaptive adolescent functioning. Adolescents reporting higher levels of parental overprotection showed more maladaptive behaviors (internalizing problems and antisocial behavior) at age 11. After controlling for parental warmth and rejection, perceived overprotection was also associated with lower levels of adaptive functioning (academic achievement) at age 11, but no longer associated with antisocial behavior. Except for internalizing problems, parental perceived overprotection was not related to longitudinal changes in adolescent functioning (antisocial behavior, prosocial behavior, and academic achievement). This longitudinal association disappeared after controlling for baseline levels of internalizing problems, suggesting that this result was less robust than expected. Moreover, mothers and fathers showed no differences in their associations between perceived overprotection and adolescent functioning. These key findings, the potential implications, and directions for future research will be discussed below.

### Concurrent Associations Between Perceived Overprotection and Adolescent Functioning

To yield a more complete picture of adolescent functioning, both maladaptive and adaptive outcomes of adolescent functioning were examined. Altogether, it was found that at age 11 perceived overprotection was positively related to maladaptive and, when controlling for related positive and negative parenting behaviors, negatively related to adaptive functioning. As such, this study confirmed many of the findings of earlier work on overprotection and maladaptive functioning (De Roo et al., 2022), but also added new knowledge on overprotection in relation to the understudied topic of adaptive functioning in adolescents.

Concurrent associations were average to small, with strongest effects for internalizing problems. As expected, adolescents who perceived their parents as overprotective experienced higher levels of internalizing problems and displayed more antisocial behaviors. The hypotheses regarding adaptive functioning were only confirmed after statistically controlling for related parental behaviors: Perceived overprotection was related to lower levels of academic achievement when controlled for parental warmth and rejection. The Self-Determination Theory suggests that children’s psychological needs (such as autonomy, competence, and relatedness) are fulfilled through positive parenting practices, such as warmth, but not met through negative parenting practices, such as rejection. Sensitivity analyses showed that parental warmth and rejection were related to initial levels and development of adolescents’ psychological, academic, and social functioning. Moreover, the results showed that the associations between perceived overprotection and maladaptive functioning were attenuated, and its associations with adaptive functioning were strengthened when accounting for other positive and negative parenting behaviors (warmth and rejection). These findings highlight that accounting for other parenting behaviors is important, as it may provide additional knowledge on the extent to which overprotection is related to adolescent functioning.

### Longer-Term Associations Between Perceived Overprotection and Adolescent Functioning

Between ages 11 and 16, adolescents, on average, showed lower levels of internalizing problems, academic achievement, prosocial behavior, and antisocial behavior. Yet, the effect sizes of the decreases were rather small. Although the decrease in internalizing problems was unexpected, additional analyses showed that this decrease was mainly observed in (all subtypes of) internalizing problems in boys. Girls showed an increase in anxious and affective problems over time. These findings are in line with research on sex differences in internalizing problems throughout adolescence, namely, that internalizing problems from childhood on continue to increase in girls, but in boys they decline from adolescence on (Gutman & McMaster, 2020). Interestingly, both boys and girls showed decreases in somatic problems. It seems that the development of internalizing problems depended more strongly on the decrease in somatic problems. Perhaps such problems are more relevant to internalizing problems in this particular age range than anxious and affective problems. This possible explanation is in line with a previous study showing that individual increases or decreases in internalizing problems depended on whether age-specific manifestations of internalizing problems were taken into account, specifically by discarding somatic problems (Petersen et al., 2018).

Theoretically, as overprotection may interfere with adolescents’ development of autonomy, parents’ efforts to protect a child from adversity, may in fact insufficiently prepare their children for adulthood (Ungar, 2009). Hence, based on theory it was expected that overprotection not only had a short-term impact (e.g., resistance of children), but its effects might last for years. The findings did not confirm these expectations: Parental perceived overprotection was found to be only related to the development of internalizing problems. Unexpectedly, higher perceived overprotection at age 11 was related to a stronger decrease in internalizing problems throughout adolescence (in both girls and boys). This finding is in contrast with ample theoretical and empirical evidence that autonomy limiting parenting, as overprotection entails, is related to more internalizing problems, especially during adolescence (McLeod et al., 2007). However, it might be that the decrease of internalizing problems over time among adolescents with relatively higher levels of parental perceived overprotection represents the statistical phenomenon known as regression to the mean. Thus, this result may reflect that adolescents who reported high levels of overprotection at T1 also experienced high levels of internalizing problems and these problems were likely to decrease over time as a result of regression to the mean. As a sensitivity analysis, this potential explanation was tested by accounting for the development of internalizing problems predicted by initial levels of internalizing problems. Although it is not a common practice in Latent Growth Curve Analyses, this method allows to control for the fact that initial levels of internalizing problems may affect how internalizing problems develop over time and to address potential regression to the mean (Lord’s Paradox; Lord, 1967). The initial finding that higher levels of perceived overprotection were associated with decreases in internalizing problems over time was no longer significant, suggesting that this result was less robust than expected.

Longer-term associations were absent for the academic and social domains of functioning in adolescents. From early adolescence on, increasingly more time is spent with friends and at school and adolescents rely more often on peers for support and advise (Soenens et al., 2019), creating ample possibilities to grow in the academic and social domain. Due to the increased influence by peers and school during adolescence, the strength of associations between parents’ overprotection and academic and social functioning might decrease during this life phase.

In general, the findings provide greater support for concurrent associations between perceived overprotection and adolescent functioning, compared with longitudinal associations. This might indicate that the consequences of overprotection for adolescent functioning may be stronger closer in time and fade out over time (De Roo et al., 2022). The behavior of the adolescent may have triggered overprotection in their parents, and as such may explain why concurrent associations were found but results did not reveal longitudinal associations. Also, parents may adjust their level of protection to more age-normative patterns when their relationship with their child transforms. Most parents gradually relinquish control and allow an increasing amount of autonomy during such transitions (Keijsers & Poulin, 2013; Soenens et al., 2019). Possible fluctuations in the levels of perceived overprotection of parents over time may have resulted in a lack of long-term associations as well. A suggestion for future research is to extend the longitudinal design by adding repeated measures of perceived overprotection to gain more insight in how parental overprotection evolves through adolescence and associates with adolescent functioning.

### Mothers’ and Fathers’ Perceived Overprotection and Adolescent Functioning

This study also differentiated between mothers’ and fathers’ perceived overprotection in relation to adolescent functioning. Mothers and fathers did not differ in their pathways from perceived overprotection to (mal)adaptive functioning in adolescents, also when accounting for parental warmth and rejection. These findings are in contrast to previous studies in which associations between overprotection and maladaptive functioning differed in mothers and fathers (e.g., De Roo et al., 2022). In these studies, parent gender differences were explained as possible differences in traditional gender roles in which mothers may play a more central role in parenting, compared to fathers (De Roo et al., 2022). Besides the lack of parent gender differences in the associations, the current study also found a high correlation between mothers’ and fathers’ perceived overprotection. Overall, these results indicate that adolescents perceived similar patterns in their parent’s overprotection, perhaps because in this sample mothers and fathers both showed relatively high levels of involvement and thus did not differ much in their gender roles. A recommendation for future research is to investigate the role of mothers’ and fathers’ involvement in investigating parent gender differences in the associations between overprotection and adolescent functioning.

### Limitations

Despite a three-wave longitudinal design among 2229 adolescents, and preregistered hypotheses, this study is not without shortcomings. Perceived overprotection was only measured at T1, which makes the conclusions of this study limited to knowledge about the associations of overprotection measured at T1 with adolescent functioning assessed at multiple times. T2 and T3 measures of perceived overprotection would have allowed to yield more insight into certain longitudinal mechanisms between overprotection and adolescent functioning. Particularly, it would have been possible to investigate how perceived overprotection developed over time and how this development is associated with different developmental domains. It would have also been possible to gain insight into whether parents show overprotective behaviors in reaction to their child’s behaviors (bidirectionality; Rapee, 1997). Future research, in which overprotection is repeatedly assessed is needed to test developmental and bidirectional mechanisms.

Even though multiple informants (adolescents and teachers) for the T3 measures were included, the use of solely questionnaires could have been strengthened by employing a multi-method study design. Overprotection was measured through one source (self-reports) only. Yet, adolescent self-reports may serve of great significance, as associations between overprotection and adolescent well-being may highly depend on the extent to which adolescents actually feel that their parents are overprotective.

Some methodological and theoretical notions should be taken into account when interpreting the results of teacher-reported prosocial behavior and academic achievement. Between T1 and T2, adolescents transitioned from elementary school to secondary school. Hence, different teachers reported on adolescents’ prosocial behavior and academic achievement throughout the study. Primary school teachers have more opportunities to get to know and to observe their students, as they are the students’ parttime or fulltime teacher. The “mentor” in secondary school (which reported at T2 and T3) meets their students a few hours per week. Thus, the results may be confounded by the fact that teachers at T1 differed in their opportunities to observe adolescents’ social interactions and academic behaviors compared to the teachers at the later assessments.

A last limitation concerns the generalizability of the results. A large proportion (approximately 90%) of the current study sample had a native Dutch background. In some cultures overprotection may be perceived as less autonomy-limiting, and more as legitimate involvement (Liga et al., 2017). Additionally, cultures differ in their views and expectations of which roles, responsibilities, and behaviors indicate that an individual has become an adult (Arnett, 2001). Hence, the study results are to be interpreted in relation to an individualistic cultural context in which increased autonomy and independency are considered to be important criteria for adulthood (Cheah et al., 2010).

## Conclusion

Longitudinal research on the role of parental overprotection in relation to adolescent functioning is limited. By examining both fathers’ and mothers’ perceived overprotection in relation to maladaptive *and* adaptive adolescent functioning, this preregistered longitudinal study showed that parental perceived overprotection is mainly concurrently and negatively related to adolescents’ functioning. Higher perceived overprotection was concurrently related to more internalizing problems and more antisocial behavior on the one hand, and lower academic achievement on the other hand (depending on the exact model). Some of the associations disappeared or emerged when adolescents’ perceived warmth and rejection from their parents were taken into account, underscoring the multidimensionality of overprotective parenting. Future research using a more extensive longitudinal design, is needed to explore the development of parental overprotection and to unravel bidirectional relations between overprotection and adolescent functioning.

## Supplementary information

Supplementary Information

Supplementary Information

## Footnotes

1 Because the slope variance of prosocial behavior was not significant, only the path from perceived overprotection to the intercept factor of prosocial behavior was included in the models (and the slope factor was constrained to zero).

2 In additional analyses adolescent sex differences were investigated regarding initial levels and development of the subtypes of internalizing problems (anxious problems, affective problems, somatic complains) (see Supplementary Material 2, Table 6). Significant group differences were found for the intercept and slope factors of all three subtypes. Girls showed higher initial levels of anxious problems, affective problems, and somatic problems, compared to boys. Girls showed an increase in anxious problems, and affective problems, whereas boys showed decreases on these subtypes of internalizing problems. For somatic problems, girls showed a stronger decrease than boys.

## References

[CR2] Achenbach TM, Rescorla LA (2001). Manual for the ASEBA school-age forms and profiles.

[CR3] Aluja A, Barrio VD, García LF (2006). Do parents and adolescents differ in their perceptions of rearing styles? Analysis of the EMBU versions for parents and adolescents. Scandinavian Journal of Psychology.

[CR4] Arnett JJ (1999). Adolescent storm and stress reconsidered. American Psychologist.

[CR5] Arnett JJ (2001). Conceptions of the transition to adulthood: Perspectives from adolescence through midlife. Journal of Adult Development.

[CR6] Arslan İB, Lucassen N, Van Lier PAC, De Haan AD, Prinzie P (2021). Early childhood internalizing problems, externalizing problems and their co-occurrence and (mal)adaptive functioning in emerging adulthood: A 16-year follow-up study. Social Psychiatry and Psychiatric Epidemiology.

[CR8] Bernstein G, Triger Z (2010). Over-parenting. UC Davis Law Review.

[CR9] Blum HP (2004). Separation-Individuation Theory and Attachment Theory. Journal of the American Psychoanalytic Association.

[CR10] Bongers IL, Koot HM, van der Ende J, Verhulst FC (2003). The normative development of child and adolescent problem behavior. Journal of Abnormal Psychology.

[CR11] Brown BB, Lerner RM, Steinberg L (2004). Adolescents’ relationships with peers. Handbook of adolescent psychology.

[CR12] Carlo G, Padilla-Walker LM, Nielson MG (2015). Longitudinal bidirectional relations between adolescents’ sympathy and prosocial behavior. Developmental Psychology.

[CR13] Cheah CSL, Trinder KM, Gokavi TN (2010). Urban/rural and gender differences among Canadian emerging adults. International Journal of Behavioral Development.

[CR14] Deković M, ten Have M, Vollebergh W, Pels T, Oosterwegel A, Wissink I, Ormel J (2006). The cross-cultural equivalence of parental rearing measure: EAABU-C. European Journal of Psychological Assessment.

[CR15] Duncan TE, Duncan SC (2004). An introduction to latent growth curve modeling. Behavior Therapy.

[CR16] Eisenberg, N., Spinrad, T.L., & Knafo-Noam, A. (2015). Prosocial development. In M.E. Lamb & R.M. Lerner (Eds.), *Handbook of child psychology and developmental science: Socioemotional processes* (Vol. 3, 7th ed., pp. 1–47). John Wiley & Sons, Inc. 10.1002/9781118963418.childpsy315

[CR17] Fagan J, Day R, Lamb ME, Cabrera NJ (2014). Should researchers conceptualize differently the dimensions of parenting for fathers and mothers?. Journal of Family Theory & Review.

[CR18] Fingerman KL, Cheng Y, Wesselmann ED, Zarit SH, Furstenberg FF, Birditt KS (2012). Helicopter parents and landing pad kids: Intense parental support of grown children. Journal of Marriage and the Family.

[CR19] Galambos, N., & Costigan, C.L. (2003). Emotional and personality development in adolescence. In I. B. Weiner (Series Ed.) & R. M. Lerner, M. A. Easterbrooks, & J. Mistry (Vol. Eds.), *Handbook of psychology: Vol. 6. Developmental psychology* (pp. 211–240). Wiley.

[CR20] Ganzeboom HBG, Treiman DJ (1996). Internationally comparable measures of occupational status for the 1988 International Standard Classification of Occupations. Social Science Research.

[CR21] Graham JW (2003). Adding missing-data-relevant variables to FIML-based structural equation models. Structural Equation Modeling.

[CR22] Gutman LM, McMaster NC (2020). Gendered pathways of internalizing problems from early childhood to adolescence and associated adolescent outcomes. Journal of Abnormal Child Psychology.

[CR23] Keijsers L, Poulin F (2013). Developmental changes in parent–child communication throughout adolescence. Developmental Psychology.

[CR24] Keijsers L, Branje S, Hawk ST, Schwartz SJ, Frijns T, Koot, Meeus W (2012). Forbidden friends as forbidden fruit: Parental supervision of friendships, contact with deviant peers, and adolescent delinquency. Child Development.

[CR25] Keyes CL, Bauer GF, Hämmig O (2014). Mental health as a complete state: How the salutogenic perspective completes the picture. Bridging occupational, organizational and public health: A transdisciplinary approach.

[CR26] Khaleque A (2013). Perceived parental warmth, and children’s psychological adjustment, and personality dispositions: A meta-analysis. Journal of Child and Family Studies.

[CR27] Kline RB (2016). Principles and Practice of Structural Equation Modeling.

[CR28] Lareau A (2011). Unequal Childhoods.

[CR29] Liga F, Ingoglia S, Inguglia C, Lo Coco A, Lo Cricchio MG, Musso P, Gutow MR (2017). Associations among psychologically controlling parenting, autonomy, relatedness, and problem behaviors during emerging adulthood. Journal of Psychology.

[CR30] Lindenberg, S. (1998). Solidarity: Its microfoundations and macro-dependence. A framing approach. In: P. Doreian & T.J. Fararo (Eds.). *The Problem of Solidarity: Theories and Models* (pp. 61–112). Gordon and Breach.

[CR31] Lord FM (1967). A paradox in the interpretation of group comparisons. Psychological Bulletin.

[CR32] Lunetti C, Iselin AMR, Di Giunta L, Lansford JE, Eisenberg N, Pastorelli C, Rothenberg WA (2022). Development of internalizing symptoms during adolescence in three countries: The role of temperamentand parenting behaviors.*European Child & Adolescence*. Psychiatry.

[CR33] Maciejewski DF, van Lier PA, Branje SJ, Meeus WH, Koot HM (2017). A daily diary study on adolescent emotional experiences: Measurement invariance and developmental trajectories. Psychological Assessment.

[CR34] Majdandžić M, de Vente W, Colonnesi C, Bögels SM (2018). Fathers’ challenging parenting behavior predicts less subsequent anxiety symptoms in early childhood. Behaviour Research and Therapy.

[CR35] Markus MTH (2003). Factors of perceived parental rearing styles: The EMBU-C examined in a sample of Dutch primary school children. Personality and Individual Differences.

[CR36] Martin AJ, Way J, Bobis J, Anderson J (2015). Exploring the ups and downs of mathematics engagement in the middle years of school. The Journal of Early Adolescence.

[CR37] McLeod BD, Wood JJ, Weisz JR (2007). Examining the association between parenting and childhood anxiety: A meta-analysis. Clinical Psychology Review.

[CR39] Moffitt TE, Silva P (1988). Self-reported delinquency. Australian and New Zealand Journal of Criminology.

[CR38] Moffitt TE, Cullen FT, Wright JP, Blevins KR (2006). A review of research on the taxonomy of life-course persistent versus adolescence-limited antisocial behavior. Taking stock: The status of criminological theory.

[CR40] Muthén LK, Muthén BO (2017). Mplus: Statistical Analysis with Latent Variables: User’s Guide (Version 8).

[CR1] National Academies of Sciences, Engineering, and Medicine. (2020). Promoting positive adolescent health behaviors and outcomes: Thriving in the 21st century.

[CR41] Oldehinkel A, Rosmalen J, Buitelaar J, Hoek H, Ormel J, Raven, Hartman C (2015). Cohort profileupdate: The TRacking Adolescents’ Individual Lives Survey (TRAILS). International Journal of Epidemiology.

[CR42] Ormel J, Oldehinkel AJ, Sijtsema J, van Oort F, Raven D, Veenstra R, Verhulst FC (2012). The TRacking Adolescents’ Individual Lives Survey (TRAILS): Design, current status, and selected findings. Journal of the American Academy of Child and Adolescent Psychiatry.

[CR43] Paquette D (2004). Theorizing the father-child relationship: Mechanisms and developmental outcomes. Human Development.

[CR44] Petersen IT, Lindhiem O, LeBeau B, Bates JE, Pettit GS, Lansford JE, Dodge KA (2018). Development of internalizing problems from adolescence to emerging adulthood: Accounting for heterotypic continuity with vertical scaling. Developmental Psychology.

[CR45] Rapee RM (1997). Potential role of childrearing practices in the development of anxiety and depression. Clinical Psychology Review.

[CR46] De Roo M, Veenstra R, Kretschmer T (2022). Internalizing and externalizing correlates of parental overprotection as measured by the EMBU: A systematic review and meta‐analysis. Social Development.

[CR47] Rote WM, Olmo M, Feliscar L (2020). Helicopter parenting and perceived overcontrol by emerging adults: A family-level profile analysis. Journal of Child and Family Studies.

[CR48] Ryan RM, Deci EL, Deci EL, Ryan RM (2002). Overview of self-determination theory: An organismic dialectical perspective. Handbook of Self-Determination Research.

[CR49] Sawilowsky SS (2009). New effect size rules of thumb. Journal of Modern Applied Statistical Methods.

[CR50] Schafer, J.L. (1997). *Analysis of incomplete multivariate data*. Chapman & Hall.

[CR51] Schiffrin HH, Liss M, Miles-McLean H, Geary KA, Erchull MJ, Tashner T (2014). Helping or hovering? The effects of helicopter parenting on college students’ well-being. Journal of Child and Family Studies.

[CR52] Segrin C, Woszidlo A, Givertz M, Montgomery N (2013). Parent and child traits associated with overparenting. Journal of Social and Clinical Psychology.

[CR53] Sentse M, Dijkstra JK, Lindenberg S, Ormel J, Veenstra R (2010). The delicate balance between parental protection, unsupervised wandering, and adolescents’ autonomy and its relation with antisocial behavior: The TRAILS study. International Journal of Behavioral Development.

[CR54] Sijtsema JJ, Verboom CE, Penninx BWJH, Verhulst FC, Ormel J (2014). Psychopathology and academic performance, social well-being, and social preference at school: The TRAILS study. Child Psychiatry and Human Development.

[CR55] Soenens B, Vansteenkiste M, Luyten P (2010). Toward a domain‐specific approach to the study of parental psychological control: Distinguishing between dependency‐oriented and achievement‐oriented psychological control. Journal of Personality.

[CR56] Soenens, B., Vansteenkiste, M., & Beyers, W. (2019). Parenting adolescents. In Marc H. Bornstein (Ed.), *Handbook of parenting: Children and parenting* (Vol. 1, 3rd ed., pp. 101–167). Routledge.

[CR57] Tremblay RE, Vitaro F, Gagnon C, Piche C (1992). A prosocial scale for the Preschool Behaviour Questionnaire: Concurrent and predictive correlates. International Journal of Behavioral Development.

[CR58] Ungar M (2009). Overprotective parenting: Helping parents provide children the right amount of risk and responsibility. The American Journal of Family Therapy.

[CR59] Veenstra R, Lindenberg S, Oldehinkel AJ, De Winter AF, Verhulst FC, Ormel J (2008). Prosocial and antisocial behavior in preadolescence: Teachers’ and parents’ perceptions of the behavior of girls and boys. International Journal of Behavioral Development.

[CR60] Verboom CE, Sijtsema JJ, Verhulst FC, Penninx BWJH, Ormel J (2014). Longitudinal associations between depressive problems, academic performance, and social functioning in adolescent boys and girls. Developmental Psychology.

[CR7] World Health Organization. (2006). Constitution of the World Health Organization.

[CR61] Westerhof G, Keyes C (2010). Mental illness and mental health: The two continua model across the lifespan. Journal of Adult Development.

[CR62] Young BJ, Wallace DP, Imig M, Borgerding L, Brown-Jacobsen AM, Whiteside SPH (2013). Parenting behaviorsand childhood anxiety: A psychometric investigation of the EMBU-C. Journal of Child and Family Studies.

