# Supplementary material for: When Too Much Help is of No Help: Mothers’ and Fathers’ Perceived Overprotective Behavior and (Mal)Adaptive Functioning in Adolescents
Source: J Youth Adolesc. 2023 Jan 12;52(5):1010–23. doi: 10.1007/s10964-022-01723-0 (PMC10027782; doi:10.1007/s10964-022-01723-0)
Supplement: Supplementary file 1 — Supplementary Information [file 10964_2022_1723_MOESM1_ESM.docx]

**Supplementary Material 1**

We computed a series of Confirmatory Factor Analyses in M*plus* 8.5 (procedure Van de Schoot & colleagues, 2012) to test whether we could confirm factor structures for mothers and fathers’ overprotection in earlier work (Aluja et al., 2006; Gerlsma et al., 1991; Laird & De Los Reyes, 2013; Laird & Weems, 2011; Someya et al., 1999). Because previous studies demonstrated that the means and variances of the latent factor would potentially differ for mothers and fathers, the latent means and variances of fathers were left unconstrained, according to the Reference-Group Method (Little et al., 2006). Syntaxes can be found through the following link: https://osf.io/r65ns/?view_only=02eb52d59001476ab61fcf63041fd9eb.

First, we tested a one-factor configural model for fathers and mothers both separately (Model 1M and 1F) to test the fit for each, and then in multigroup mode (Model 1MG) to use as comparison for invariance tests. Table 2 shows that although the RSMEAs were sufficient (.04-.05), the configural models had a relatively poor fit for mothers and fathers based on the other indices. For the multigroup configural model of fathers and mothers, the standardized factor loadings were sufficient (average .41 for mothers and .42 for fathers, ranging from .30 to .51 for mothers, and from .28 to .49 for fathers). Overall, the findings replicate the one factor structure of overprotection in both mothers and fathers.

Next, we examined differences in the model between fathers and mothers, by testing whether the fit would change meaningfully (∆CFI = ≥.01, ΔRMSEA, ≥.0.015; Chen, 2007) by adding equality constraints between fathers and mothers on factor loadings (testing metric invariance, Model 2), and both factor loadings and intercepts (testing scalar invariance, Model 3). Compared with the configural multigroup model (Model 1MG), none of the constraints significantly changed the fit in terms of both CFI and RMSEA. Therefore, the underlying model was configural invariant, which suggests that the underlying factor structure is similar for fathers and mothers. This similar factor structure of the overprotection scale allows comparisons between fathers and mothers.

**References**

Aluja, A., Barrio, V. D., & García, L. F. (2006). Do parents and adolescents differ in their perceptions of rearing styles? Analysis of the EMBU versions for parents and adolescents. *Scandinavian Journal of Psychology, 47*(2), 103-108. https://doi.org/10.1111/j.1467-9450.2006.00497.x

Gerlsma, C., Arrindell, W. A., Van der Veen, N., & Emmelkamp, P. M. (1991). A parental

rearing style questionnaire for use with adolescents: Psychometric evaluation of the EMBU-A. *Personality and Individual differences, 12*(12), 1245-1253. https://doi.org/10.1016/0191-8869(91)90196-I

Laird, R. D., & Weems, C. F. (2011). The equivalence of regression models using difference scores and models using separate scores for each informant: Implications for the study of informant discrepancies. *Psychological Assessment, 23*(2), 388-397. https://doi.org/10.1037/a0021926

Little, T. D., Slegers, D. W., & Card, N. A. (2006). A non-arbitrary method of identifying and

scaling latent variables in SEM and MACS models. Structural Equation Modeling, 13(1), 59–72. https://doi.org/10.1207/s15328007sem1301_3

Muthén, B., & Satorra, A. (1995). Complex sample data in structural 1070 equation modeling.

*Sociological Methodology, 25,* 267. https://doi.org/10.2307/271070

Someya, T., Uehara, T., Kadowaki, M., Sakado, K., Reist, C., Tang, S. W., & Takahashi, S. (1999). Factor analysis of the EMBU scale in a large sample of Japanese volunteers. *Acta Psychiatrica Scandinavica, 100*(4), 252-257. https://doi.org/10.1111/j.1600-0447.1999.tb10858.x

Van de Schoot, R., Lugtig, P., & Hox, J. (2012). A checklist for testing 1154 measurement

invariance. *European Journal of Developmental Psychology, 9*(4), 486–492. https://doi.org/10.1080/17405629.2012.686740

**Table 1.**

*Descriptives for Mothers’ and Fathers’ Overprotection, Rejection, and Warmth (T1).*

|  | *M*(*SD)* | Range | *N* |
| --- | --- | --- | --- |
| Mothers’ Overprotection | 1.93(0.41) | 1.00-3.75 | 2,193 |
| Fathers’ Overprotection | 1.79(0.39) | 1.00-3.50 | 2,141 |
| Mothers’ Rejection | 1.48(0.33) | 1.00-3.94 | 2,193 |
| Fathers’ Rejection | 1.48(0.34) | 1.00-3.59 | 2,140 |
| Mothers’ Warmth | 3.28(0.49) | 1.06-4.00 | 2,194 |
| Fathers’ Warmth | 3.15(0.56) | 1.00-4.00 | 2,142 |

*Note.* Significant mean level differences were found for mothers’ and fathers’ overprotection (*t*(2128) =

-26.18, *p* < .001) and warmth (*t*(2129) = -17.95, *p* < .001) (paired t-tests).

**Table 2.**

|  | *χ2 (df)* | CFI | | TLI | RMSEA[CI] | ∆CFI | ∆RMSEA |
| --- | --- | --- | --- | --- | --- | --- | --- |
| Model 1M: Configural Model Mothers | 272.74(54) | .888 | .863 | | .04[.04, .05] | - | - |
| Model 1F: Configural Model Fathers | 345.34(54) | .866 | .836 | | .05[.05, .06] | - | - |
| Model 1MG: Configural Model Multigroup | 698.48(118) | .859 | .842 | | .05[.04, .05] | - | - |
| Model 2: Metric Invariance | 666.82(120) | .867 | .854 | | .05[.04, .05] | .01^a^ | .00^a^ |
| Model 3: Scalar Invariance | 901.92(132) | .813 | .813 | | .05[.05, .06] | .03^a^ | .00^a^ |

*Test for Invariance of Measures: Goodness-of-Fit Statistics.*

*Note.* CFI = Comparative Fit Index; RMSEA = Root Mean Squared Error of Approximation; TLI = Tucker-Lewis Index. ^a^ Compared with Configural Model Multigroup.

**Supplementary Material 2**

**Table 1.**

|  | T1 | | | | T2 | | | | T3 | | | |
| --- | --- | --- | --- | --- | --- | --- | --- | --- | --- | --- | --- | --- |
|  | Internalizing problems | Academic Achievement | Prosocial Behavior | Antisocial Behavior | Internalizing problems | Academic Achievement | Prosocial Behavior | Antisocial Behavior | Internalizing problems | Academic Achievement | Prosocial Behavior | Antisocial Behavior |
| Warmth | -.15^**^ | .22^**^ | .19^**^ | -.22^**^ | -.08^**^ | .12^**^ | .15^**^ | -.10^**^ | -.05^*^ | .07^*^ | .12^**^ | -.08^**^ |
| Rejection | .41^**^ | -.11^**^ | -.13^**^ | .31^**^ | .22^**^ | -.09^**^ | -.06^*^ | .12^**^ | .15^**^ | -.02 | -.02 | .10^**^ |
| SES | -.05^*^ | -.36^**^ | .28^**^ | -.14^**^ | -.04 | .18^**^ | .17^**^ | -.12^**^ | -.08^**^ | .12^**^ | .22^**^ | -.15^**^ |
| Age | -.05^*^ | -.03 | -.004 | .13^**^ | -.03 | -.02 | -.04 | .07^**^ | -.01 | -.02 | .05 | -.02 |

*Bivariate Correlations Between (Background) Covariates and (Mal)Adaptive Functioning in Adolescence.*

Note. * p < .05. ** p < .01. *** p < .001.

**Table 2.**

*Linear Growth Curve Models: Goodness-of-Fit Statistics.*

|  | *χ2(df)* | CFI | TLI | RMSEA[CI] | |
| --- | --- | --- | --- | --- | --- |
| *Research Question 1: Parental Perceived Overprotection and Adolescent Functioning* | | | | |  |
| Internalizing Problems | 4.03(1) | .997 | .990 | .04[.01, .08] | |
| Academic Achievement | 15.19(1) | .935 | .806 | .08[.05, .12] | |
| Prosocial Behavior | 2.43(1) | .995 | .985 | .03[.00, .07] | |
| Antisocial Behavior | 0.01(1) | 1.00 | 1.00 | .00[.00, .01] | |
| Overprotection 🡪 Internalizing Problems | 23.00(7) | .990 | .971 | .03[.02, .05] | |
| Overprotection 🡪 Academic Achievement | 29.94(7) | .963 | .890 | .04[.03, .06] | |
| Overprotection 🡪 Prosocial Behavior | 35.45(15) | .973 | .962 | .03[.02, .04] | |
| Overprotection 🡪 Antisocial Behavior | 20.82(7) | .988 | .963 | .03[.02, .05] | |
| *Research Question 2: Parental Gender Differences: Maternal and Paternal Perceived Overprotection* | | | | |  |
| Unconstrained Model: Overprotection 🡪 Internalizing Problems | 42.34(14) | .991 | .973 | .03[.02, .04] | |
| Constrained Model: Overprotection 🡪 Internalizing Problems | 42.06(16) | .992 | .979 | .03[.02, .04] | |
| Unconstrained Model: Overprotection 🡪 Academic Achievement | 61.10(14) | .961 | .884 | .04[.03, .05] | |
| Constrained Model: Overprotection 🡪 Academic Achievement | 62.69(16) | .962 | .899 | .04[.03, .05] | |
| Unconstrained Model: Overprotection 🡪 Prosocial Behavior | 70.30(30) | .973 | .962 | .03[.02, .03] | |
| Constrained Model: Overprotection 🡪 Prosocial Behavior | 70.85(31) | .973 | .963 | .03[.02, .03] | |
| Unconstrained Model: Overprotection 🡪 Antisocial Behavior | 39.10(14) | .989 | .966 | .03[.02, .04] | |
| Constrained Model: Overprotection 🡪 Antisocial Behavior | 39.45(16) | .989 | .972 | .03[.02, .04] | |

*Note.* CFI = Comparative Fit Index; RMSEA = Root Mean Squared Error of Approximation; TLI = Tucker-Lewis Index. CI = 90% Confidence Interval.

**Table 3.**

*Sensitivity Analyses With Covariates Parental Warmth and Rejection: Goodness-of-Fit Statistics.*

|  | *χ2(df)* | CFI | TLI | RMSEA[CI] |
| --- | --- | --- | --- | --- |
| *Research Question 1: Parental Perceived Overprotection and Adolescent Functioning* | | | |  |
| Overprotection 🡪 Internalizing Problems | 26.23(9) | .991 | .972 | .03[.02, .04] |
| Overprotection 🡪 Academic Achievement | 31.84(9) | .966 | .899 | .04[.02, .05] |
| Overprotection 🡪 Prosocial Behavior | 48.13(19) | .963 | .948 | .03[.02, .04] |
| Overprotection 🡪 Antisocial Behavior | 33.12(9) | .981 | .944 | .04[.02, .05] |
| *Research Question 2: Parental Gender Differences: Maternal and Paternal Perceived Overprotection* | | | | |
| Unconstrained Model: Overprotection 🡪 Internalizing Problems | 47.57(18) | .992 | .975 | .03[.02, .04] |
| Constrained Model: Overprotection 🡪 Internalizing Problems | 47.60(20) | .992 | .979 | .03[.02, .04] |
| Unconstrained Model: Overprotection 🡪 Academic Achievement | 64.91(18) | .964 | .893 | .04[.03, .05] |
| Constrained Model: Overprotection 🡪 Academic Achievement | 69.11(20) | .963 | .899 | .04[.03, .04] |
| Unconstrained Model: Overprotection 🡪 Prosocial Behavior | 91.28(38) | .966 | .951 | .03[.02, .03] |
| Constrained Model: Overprotection 🡪 Prosocial Behavior | 92.37(39) | .966 | .952 | .03[.02, .03] |
| Unconstrained Model: Overprotection 🡪 Antisocial Behavior | 60.27(18) | .983 | .949 | .03[.02, .04] |
| Constrained Model: Overprotection 🡪 Antisocial Behavior | 60.28(20) | .984 | .956 | .03[.02, .04] |

*Note.* CFI = Comparative Fit Index; RMSEA = Root Mean Squared Error of Approximation; TLI = Tucker-Lewis Index. CI = 90% Confidence Interval.

**Table 4**.

*Parameter Estimates of the Regression Paths From Covariates Warmth and Rejection at T1 to (Mal)Adaptive Functioning in Adolescence, from Main Models with Perceived Overprotection.*

|  | Internalizing Problems | | Academic Achievement | | | Prosocial Behavior | | | Antisocial Behavior | | |
| --- | --- | --- | --- | --- | --- | --- | --- | --- | --- | --- | --- |
|  | B(*SE*) | *β* | B(*SE*) | *β* | B(*SE*) | | *β* | B(*SE*) | | *β* |  |
| Warmth _🡪_  Intercept | -.05(.01)^***^ | -.12 | .29(.04)^***^ | .25 | .14(.03)^***^ | | .17 | -.08(.02)^***^ | | -.13 |  |
| Warmth  _🡪_ Slope | .01(.01) | .04 | -.13(.04)^***^ | -.24 | N/A | | N/A | .03(.01)^**^ | | .11 |  |
| Rejection _🡪_ Intercept | .23(.02)^***^ | .37 | -.03(.08) | -.02 | .02(.04) | | .02 | .24(.04)^***^ | | .24 |  |
| Rejection _🡪_ Slope | -.07(.01)^***^ | -.23 | .05(.06) | .06 | N/A | | N/A | -.12(.02)^***^ | | -.29 |  |

Note. ** p < .01. *** p < .001. **Table 5**.

*Parameter Estimates of the Intercept and Slope Factors for the Linear Growth Model and the Regression Paths From Overprotection to Internalizing Problems in Adolescence, for Girls and Boys.*

|  | Internalizing problems | | | | |
| --- | --- | --- | --- | --- | --- |
|  | Girls | | Boys | |  |
|  | *M(SE)* | | *M(SE)* | | Δχ^2^(*df) ^a^* |
| Overall model |  | |  | | 231.55(2)^***^ |
| Intercept factor | .39(.01)^***^ | | .33(.01)^***^ | | 33.37(1)^***^ |
| Slope factor | -.001(.01) | | -.06(.004)^***^ | | 82.36(1)^***^ |
|  | B(*SE*) | *β* | B(*SE*) | *β* | Δχ^2^(*df) ^a^* |
| Overall model |  |  |  |  | 2.76(2) |
| Perceived overprotection _🡪_  Intercept | .22(.02)^***^ | .41 | .18(.02)^***^ | .40 | 5.44(1)^*^ |
| Perceived overprotection _🡪_ Slope | -.06(.01)^***^ | -.20 | -.05(.01)^***^ | -.25 | 3.09(1) |

Note. ^a^ Comparison of Freely Estimated Model Versus Constrained Model (Chi-Square Difference Test).

**p* < .05, *** *p* < .001.

**Table 6**.

*Parameter Estimates of the Intercept and Slope Factors for the Linear Growth Model of the three Subtypes of Internalizing Problems, for Girls and Boys.*

|  | Anxious problems | | | Affective problems | | | | | Somatic complaints | | | | |
| --- | --- | --- | --- | --- | --- | --- | --- | --- | --- | --- | --- | --- | --- |
|  | Girls | Boys |  | Girls | Boys |  | | Girls | | Boys |  | | |
|  | *M(SE)* | *M(SE)* | Δχ^2^(*df) ^a^* | *M(SE)* | *M(SE)* | Δχ^2^(*df) ^a^* | | | *M(SE)* | *M(SE)* | | Δχ^2^(*df) ^a^* | |
| Overall model |  |  | 173.61(2)^***^ |  |  | 154.91(2)^***^ |  | | |  | | | 155.29(2)^***^ |
| Intercept factor | .39(.01)^***^ | .32(.01)^***^ | 31.59(1)^***^ | .30(.01)^***^ | .27(.01)^***^ | 5.04(1)^*^ | .48(.01)^***^ | | | .40(.01)^***^ | | | 31.20(1)^***^ |
| Slope factor | .02(.01)^*^ | -.04(.01)^***^ | 42.52(1)^***^ | .03(.01)^***^ | -.03(.004)^***^ | 99.39(1)^***^ | -.08(.01)^***^ | | | -.12(.01)^***^ | | | 20.44(1)^***^ |

Note. ^a^ Comparison of Freely Estimated Model Versus Constrained Model (Chi-Square Difference Test).

* p < .05. *** p < .001.
