# Supplementary material for: When Too Much Help is of No Help: Mothers’ and Fathers’ Perceived Overprotective Behavior and (Mal)Adaptive Functioning in Adolescents
Source: J Youth Adolesc. 2023 Jan 12;52(5):1010–23. doi: 10.1007/s10964-022-01723-0 (PMC10027782; doi:10.1007/s10964-022-01723-0)
Supplement: Supplementary file 2 — Supplementary Information [file 10964_2022_1723_MOESM2_ESM.pdf]

Supplementary Material 1

We computed a series of Confirmatory Factor Analyses in M*plus* 8.5 (procedure Van de Schoot & colleagues, 2012) to test whether we could confirm factor structures for mothers and fathers' overprotection in earlier work (Aluja et al., 2006; Gerlsma et al., 1991; Laird & De Los Reyes, 2013; Laird & Weems, 2011; Someya et al., 1999). Because previous studies demonstrated that the means and variances of the latent factor would potentially differ for mothers and fathers, the latent means and variances of fathers were left unconstrained, according to the Reference-Group Method (Little et al., 2006). Syntaxes can be found through the following link: https://osf.io/r65ns/?view_only=02eb52d59001476ab61fcf63041fd9eb.

First, we tested a one-factor configural model for fathers and mothers both separately (Model 1M and 1F) to test the fit for each, and then in multigroup mode (Model 1MG) to use as comparison for invariance tests. Table 2 shows that although the RSMEAs were sufficient (.04-.05), the configural models had a relatively poor fit for mothers and fathers based on the other indices. For the multigroup configural model of fathers and mothers, the standardized factor loadings were sufficient (average .41 for mothers and .42 for fathers, ranging from .30 to .51 for mothers, and from .28 to .49 for fathers). Overall, the findings replicate the one factor structure of overprotection in both mothers and fathers.

Next, we examined differences in the model between fathers and mothers, by testing whether the fit would change meaningfully (ΔCFI = $\geq$.01, ΔRMSEA, $\geq$.0.015; Chen, 2007) by adding equality constraints between fathers and mothers on factor loadings (testing metric invariance, Model 2), and both factor loadings and intercepts (testing scalar invariance, Model 3). Compared with the configural multigroup model (Model 1MG), none of the constraints significantly changed the fit in terms of both CFI and RMSEA. Therefore, the underlying model was configural invariant, which suggests that the underlying factor structure is similar for fathers

and mothers. This similar factor structure of the overprotection scale allows comparisons between fathers and mothers.

Table 1.

Descriptives for Mothers' and Fathers' Overprotection, Rejection, and Warmth (T1).

	M(*SD*)	Range	*N*
Mothers' Overprotection	1.93(0.41)	1.00-3.75	2,193
Fathers' Overprotection	1.79(0.39)	1.00-3.50	2,141
Mothers' Rejection	1.48(0.33)	1.00-3.94	2,193
Fathers' Rejection	1.48(0.34)	1.00-3.59	2,140
Mothers' Warmth	3.28(0.49)	1.06-4.00	2,194
Fathers' Warmth	3.15(0.56)	1.00-4.00	2,142

Note. Significant mean level differences were found for mothers' and fathers' overprotection ($t(2128) = -26.18$, $p < .001$) and warmth ($t(2129) = -17.95$, $p < .001$) (paired t-tests).

Table 2.

Test for Invariance of Measures: Goodness-of-Fit Statistics.

	$\chi 2$ *(df)*	CFI	TLI	RMSEA[CI]	ΔCFI	ΔRMSEA
Model 1M: Configural Model Mothers	272.74(54)	.888	.863	.04[.04, .05]	-	-
Model 1F: Configural Model Fathers	345.34(54)	.866	.836	.05[.05, .06]	-	-
Model 1MG: Configural Model Multigroup	698.48(118)	.859	.842	.05[.04, .05]	-	-
Model 2: Metric Invariance	666.82(120)	.867	.854	.05[.04, .05]	.01[a]	.00[a]
Model 3: Scalar Invariance	901.92(132)	.813	.813	.05[.05, .06]	.03[a]	.00[a]

Note. CFI = Comparative Fit Index; RMSEA = Root Mean Squared Error of Approximation; TLI = Tucker-Lewis Index. [a] Compared with Configural Model Multigroup.

Supplementary Material 2

Table 1.

Bivariate Correlations Between (Background) Covariates and (Mal)Adaptive Functioning in Adolescence.

	T1				T2				T3			
	Internalizing problems	Academic Achievement	Prosocial Behavior	Antisocial Behavior	Internalizing problems	Academic Achievement	Prosocial Behavior	Antisocial Behavior	Internalizing problems	Academic Achievement	Prosocial Behavior	Antisocial Behavior
Warmth	-.15**	.22**	.19**	-.22**	-.08**	.12**	.15**	-.10**	-.05*	.07*	.12**	-.08**
Rejection	.41**	-.11**	-.13**	.31**	.22**	-.09**	-.06*	.12**	.15**	-.02	-.02	.10**
SES	-.05*	-.36**	.28**	-.14**	-.04	.18**	.17**	-.12**	-.08**	.12**	.22**	-.15**
Age	-.05*	-.03	-.004	.13**	-.03	-.02	-.04	.07**	-.01	-.02	.05	-.02

Note. * p < .05. ** p < .01. *** p < .001.

Table 2.

Linear Growth Curve Models: Goodness-of-Fit Statistics.

	χ2 (df)	CFI	TLI	RMSEA[CI]
Research Question 1: Parental Perceived Overprotection and Adolescent Functioning				
Internalizing Problems	4.03(1)	.997	.990	.04[.01, .08]
Academic Achievement	15.19(1)	.935	.806	.08[.05, .12]
Prosocial Behavior	2.43(1)	.995	.985	.03[.00, .07]
Antisocial Behavior	0.01(1)	1.00	1.00	.00[.00, .01]
Overprotection → Internalizing Problems	23.00(7)	.990	.971	.03[.02, .05]
Overprotection → Academic Achievement	29.94(7)	.963	.890	.04[.03, .06]
Overprotection → Prosocial Behavior	35.45(15)	.973	.962	.03[.02, .04]
Overprotection → Antisocial Behavior	20.82(7)	.988	.963	.03[.02, .05]
Research Question 2: Parental Gender Differences: Maternal and Paternal Perceived Overprotection				
Unconstrained Model: Overprotection → Internalizing Problems	42.34(14)	.991	.973	.03[.02, .04]
Constrained Model: Overprotection → Internalizing Problems	42.06(16)	.992	.979	.03[.02, .04]
Unconstrained Model: Overprotection → Academic Achievement	61.10(14)	.961	.884	.04[.03, .05]
Constrained Model: Overprotection → Academic Achievement	62.69(16)	.962	.899	.04[.03, .05]
Unconstrained Model: Overprotection → Prosocial Behavior	70.30(30)	.973	.962	.03[.02, .03]
Constrained Model: Overprotection → Prosocial Behavior	70.85(31)	.973	.963	.03[.02, .03]
Unconstrained Model: Overprotection → Antisocial Behavior	39.10(14)	.989	.966	.03[.02, .04]
Constrained Model: Overprotection → Antisocial Behavior	39.45(16)	.989	.972	.03[.02, .04]

Note. CFI = Comparative Fit Index; RMSEA = Root Mean Squared Error of Approximation; TLI = Tucker-Lewis Index. CI = 90% Confidence Interval.

Table 3.

Sensitivity Analyses With Covariates Parental Warmth and Rejection: Goodness-of-Fit Statistics.

	χ2 (df)	CFI	TLI	RMSEA[CI]
Research Question 1: Parental Perceived Overprotection and Adolescent Functioning				
Overprotection → Internalizing Problems	26.23(9)	.991	.972	.03[.02, .04]
Overprotection → Academic Achievement	31.84(9)	.966	.899	.04[.02, .05]
Overprotection → Prosocial Behavior	48.13(19)	.963	.948	.03[.02, .04]
Overprotection → Antisocial Behavior	33.12(9)	.981	.944	.04[.02, .05]
Research Question 2: Parental Gender Differences: Maternal and Paternal Perceived Overprotection				
Unconstrained Model: Overprotection → Internalizing Problems	47.57(18)	.992	.975	.03[.02, .04]
Constrained Model: Overprotection → Internalizing Problems	47.60(20)	.992	.979	.03[.02, .04]
Unconstrained Model: Overprotection → Academic Achievement	64.91(18)	.964	.893	.04[.03, .05]
Constrained Model: Overprotection → Academic Achievement	69.11(20)	.963	.899	.04[.03, .04]
Unconstrained Model: Overprotection → Prosocial Behavior	91.28(38)	.966	.951	.03[.02, .03]
Constrained Model: Overprotection → Prosocial Behavior	92.37(39)	.966	.952	.03[.02, .03]
Unconstrained Model: Overprotection → Antisocial Behavior	60.27(18)	.983	.949	.03[.02, .04]
Constrained Model: Overprotection → Antisocial Behavior	60.28(20)	.984	.956	.03[.02, .04]

Note. CFI = Comparative Fit Index; RMSEA = Root Mean Squared Error of Approximation; TLI = Tucker-Lewis Index. CI = 90% Confidence Interval.

Table 4.

Parameter Estimates of the Regression Paths From Covariates Warmth and Rejection at T1 to (Mal)Adaptive Functioning in Adolescence, from Main Models with Perceived Overprotection.

	Internalizing Problems		Academic Achievement		Prosocial Behavior		Antisocial Behavior	
	B(*SE*)	β	B(*SE*)	β	B(*SE*)	β	B(*SE*)	β
Warmth → Intercept	-.05(.01)***	-.12	.29(.04)***	.25	.14(.03)***	.17	-.08(.02)***	-.13
Warmth → Slope	.01(.01)	.04	-.13(.04)***	-.24	N/A	N/A	.03(.01)**	.11
Rejection → Intercept	.23(.02)***	.37	-.03(.08)	-.02	.02(.04)	.02	.24(.04)***	.24
Rejection → Slope	-.07(.01)***	-.23	.05(.06)	.06	N/A	N/A	-.12(.02)***	-.29

Note. ** p < .01. *** p < .001.

Table 5.

Parameter Estimates of the Intercept and Slope Factors for the Linear Growth Model and the Regression Paths From Overprotection to Internalizing Problems in Adolescence, for Girls and Boys.

	Internalizing problems				
	Girls		Boys		
	M(SE)		*M(SE)*		$\Delta\chi^2(df)$ [a]
Overall model					231.55(2)***
Intercept factor	.39(.01)***		.33(.01)***		33.37(1)***
Slope factor	-.001(.01)		-.06(.004)***		82.36(1)***
	B(*SE*)	β	B(*SE*)	β	$\Delta\chi^2(df)$ [a]
Overall model					2.76(2)
Perceived overprotection → Intercept	.22(.02)***	.41	.18(.02)***	.40	5.44(1)*
Perceived overprotection → Slope	-.06(.01)***	-.20	-.05(.01)***	-.25	3.09(1)

Note. [a] Comparison of Freely Estimated Model Versus Constrained Model (Chi-Square Difference Test).
*$p < .05$, *** $p < .001$.

Table 6.

Parameter Estimates of the Intercept and Slope Factors for the Linear Growth Model of the three Subtypes of Internalizing Problems, for Girls and Boys.

	Anxious problems			Affective problems			Somatic complaints		
	Girls	Boys		Girls	Boys		Girls	Boys	
	M(SE)	*M(SE)*	$\Delta\chi^2(df)^a$	*M(SE)*	*M(SE)*	$\Delta\chi^2(df)^a$	*M(SE)*	*M(SE)*	$\Delta\chi^2(df)^a$
Overall model			173.61(2)***			154.91(2)***			155.29(2)***
Intercept factor	.39(.01)***	.32(.01)***	31.59(1)***	.30(.01)***	.27(.01)***	5.04(1)*	.48(.01)***	.40(.01)***	31.20(1)***
Slope factor	.02(.01)*	-.04(.01)***	42.52(1)***	.03(.01)***	-.03(.004)***	99.39(1)***	-.08(.01)***	-.12(.01)***	20.44(1)***

Note. [a] Comparison of Freely Estimated Model Versus Constrained Model (Chi-Square Difference Test).
* p < .05. *** p < .001.
